# Gender-specific association of metabolic syndrome and its components with arterial stiffness in the general Chinese population

**DOI:** 10.1371/journal.pone.0186863

**Published:** 2017-10-26

**Authors:** Mengjia Yue, Hongjian Liu, Minfu He, Fangyuan Wu, Xuanxuan Li, Yingxin Pang, Xiaodi Yang, Ge Zhou, Juan Ma, Meitian Liu, Ping Gong, Jinghua Li, Xiumin Zhang

**Affiliations:** 1 Department of Epidemiology and Biostatistics, School of Public Health, Jilin University, Changchun, China; 2 Department of Social Medicine and Health Management, School of Public Health, Jilin University, Changchun, China; Beijing Key Laboratory of Diabetes Prevention and Research, CHINA

## Abstract

**Objectives:**

Metabolic syndrome (MS) is considered to be a cluster of interrelated risk factors for metabolism, which may increase arterial stiffness and cardiovascular morbidity. The cardio-ankle vascular index (CAVI) is a reliable indicator of arterial stiffness and early arteriosclerosis. The main objective of this study is to evaluate the gender-specific relationship between MS and CAVI in the general Chinese population.

**Methods:**

A total of 1,301 subjects aged 20 to 60 years participated in this study. CAVI was measured noninvasively using a Vasera VS-1000 device. Blood samples and waist circumference were examined to identify metabolic syndrome according to the criteria set forth in the 2009 Joint Scientific Statement.

**Results:**

The prevalence of MS in the study subjects was 17.4% (30.7% in males and 7.0% in females, P < 0.001). CAVI values were significantly higher in MS subjects than in non-MS subjects and increased linearly as the number of MS components increased in females, but not in males. Using multiple regression analysis, we found that BMI was correlated with CAVI in the overall population and in both genders, and that high-density lipoprotein cholesterol (HDL-C) was associated with CAVI in males, while the number of MS components was related to CAVI in females. CAVI values increased linearly with age in both genders (P-trend < 0.001 for both), and this correlation was stronger in males than in females.

**Conclusions:**

There are gender-specific differences in the association of MS and CAVI. First, the effects of the number of MS components on CAVI are stronger in females than in males. Second, the effect of each MS component on arterial stiffness may differ in relation to gender. In addition, aging affects arterial stiffness more severely in males, and the increase in arterial stiffness tends to occur at a younger age in males than in females. Larger samples and longitudinal studies are needed to further confirm our results in the future.

## Introduction

Metabolic syndrome (MS) is considered to be a cluster of risk factors that are interrelated with metabolism, including obesity, hypertension, dyslipidemia, and dysglycemia, which could lead to diabetes mellitus and cardiovascular disease [1, 2]. Currently, about one-quarter to one-third of adults have metabolic syndrome, and its prevalence continues to increase [3, 4]. Research has shown that the morbidity and mortality of cardiovascular disease caused by MS are significantly higher than that of healthy people and people who have a single metabolic risk factor [5].

It has been reported that there are some changes in arterial elasticity in MS patients before the occurrence by target organ damage of cardiovascular disease [6], while increased arterial stiffness plays an important role in the progress of atherosclerosis. These findings indicate that atherosclerosis may be caused partly by the clustering of MS components.

As a noninvasive and effective tool, pulse wave velocity (PWV) has been used widely in the examination of early atherosclerosis. However, it is strongly dependent on blood pressure, which leads to poor reproducibility [7]. In contrast, the new index, the cardio-ankle vascular index (CAVI), which was developed based on stiffness parameter β and PWV, has been reported as having a good predictive value for early arteriosclerosis and have much less dependence on blood pressure than PWV. Furthermore, it has been demonstrated that CAVI is a reliable indicator of arterial stiffness and early arteriosclerosis [8, 9].

As mentioned above, dyslipidemia is one of the components of MS, which is also a significant risk factor for atherosclerosis and coronary heart disease [10, 11]. Recently, researchers found that serum lipid ratios are more effective in predicting the risk of coronary artery disease (CAD) than is a single serum lipid level [12, 13]. Among them, TG / HDL-C and non-HDL-C / HDL-C have been proven to be correlated independently with arterial stiffness and CAD after adjusting for traditional risk factors [14, 15]. Furthermore, the ratio of non-HDL-C / HDL-C and the logarithm of TG / HDL-C have been used as markers of plasma atherogenicity, which was defined as the atherogenic coefficient (AC) and the atherogenic index of plasma (AIP), respectively [16, 17].

Metabolic syndrome is related to an increase in arterial stiffness, which could be detected by CAVI. Many studies have discussed the relationship between MS and arterial stiffness in other countries [18–20]. However, there has been only one study on the association of metabolic syndrome and CAVI in the middle-aged and elderly population in China [21]. Therefore, the main objective of this study was to evaluate the relationship between MS and CAVI in the general Chinese population by gender. Furthermore, two indexes of plasma atherogenicity, AC and AIP, were used along with CAVI to analyze the association of MS with arterial stiffness from another perspective.

## Methods

### Subjects

From June 2016 to October 2016, 1301 subjects (573 males and 728 females, aged 20 to 60 years) who underwent health check-ups at the First Affiliated Hospital of Jilin University (China) were recruited.

### Ethical standards

This study was reviewed and approved by the Ethics Committee of the Jilin University School of Public Health (No. 2015-12-08), and written informed consent was obtained from all subjects before they participated in the study.

### Physical and biochemical measurements

Subjects were required to avoid drinking alcohol and consuming high-fat or high-sugar foods the day before the physical assessment. Fasting blood samples were collected between 7 and 8 AM. The samples were centrifuged and analyzed for total cholesterol (TC), triglycerides (TG), HDL-C and fasting plasma glucose (FPG). AC and AIP were calculated by the following formulas [16, 22]:

$$\mathrm{AC}=\frac{\mathrm{TC-HDL-C}}{\mathrm{HDL-C}}$$

Where (TC − HDL − C = Non − HDL − C [23].)

$$\mathrm{AIP}=\mathrm{Log}\left(\frac{\mathrm{TG}}{\mathrm{HDL-C}}\right)$$

Waist circumference (WC) was measured at the midsection between the lowest rib and the iliac crest. Height and weight were measured to obtain the body mass index (BMI): BMI = weight (kg) / height (m^2^). Blood pressure was measured using an automatic blood pressure monitor, with each subject sitting for at least 5 minutes.

In addition, all participants were asked to complete a short questionnaire about personal information, their habits of smoking and drinking, their level of self-perceived life stress, and self-reported disease history of cardiovascular disease (including coronary heart disease and stroke).

### Measurement of CAVI

We used a Vasera VS-1000 system (Fukuda Denshi, Tokyo, Japan) to noninvasively measure CAVI. CAVI values were obtained by substituting the stiffness parameter β into the following equation [8, 9]:

$$\mathrm{CVAI}=a\left\{2\rho /\Delta P\left[\mathrm{In}\;\mathrm{Ps}/\mathrm{Pd}\right]{\mathrm{PWV}}^{2}\right\}+b$$

(ρ is blood density; Ps is systolic blood pressure (SBP); Pd is diastolic blood pressure (DBP); ΔP = Ps—Pd; a and b are constants; PWV is pulse wave velocity.)

The subjects were required to rest in the supine position, and the cuffs were wrapped around both their upper arms and ankles. Electrocardiographic electrodes were fixed at the wrists, and microphones were placed on the sternal angle to record an electrocardiogram and a phonocardiogram. After approximately 5 minutes, we obtained the values of CAVI and other waveforms from the Vasera VS-1000 system’s embedded printer.

### Diagnostic criteria of metabolic syndrome

According to the Joint Scientific Statement of the International Diabetes Federation; the National Heart, Lung, and Blood Institute; the American Heart Association and three other international institutions in 2009 [24], MS should be diagnosed as the presence of three or more of the following items:

1. abdominal obesity: the WC threshold for abdominal obesity in the Chinese population is 85 cm and above in males, or 80 cm and above in females;
2. a high TG level (≥ 1.7 mmol/L) or specific treatment for this dyslipidemia;
3. a low HDL-C level (≤ 1.0 mmol/L in male, ≤ 1.3 mmol/L in female), or specific treatment for this dyslipidemia;
4. raised blood pressure (SBP ≥ 130 mmHg and/or DBP ≥ 85 mmHg) or drug treatment for hypertension;
5. elevated FPG level (≥ 5.6 mmol/L) or drug treatment for elevated glucose.

### Statistical analysis

SPSS Statistics for Windows, version 20.0 (IBM Corp, Armonk, New York, USA), was used to perform the statistical analyses. The data were presented as the mean ± standard deviation (SD) or median (interquartile range). Differences in quantitative variables between two groups were evaluated using Student’s unpaired t-test, while differences in percentage of subjects were determined by *χ*^*2*^-test. One-way analysis of variance (ANOVA) was performed to detect significant difference among the groups satisfying different numbers of MS components. Post hoc analysis was conducted by Fisher’s protected least significant difference (PLSD). The relationships between MS components and CAVI were assessed by Pearson’s correlation analysis. In the multivariate analysis, multiple linear regression models were conducted (ENTER method) using CAVI values as the dependent variable and MS and its components as independent variables, using age, smoking and drinking status, high-stress life, BMI, non-HDL, coronary heart disease, and stroke as adjusting variables. *P* < 0.05 was considered statistically significant.

## Results

### Baseline characteristics of the subjects

The characteristics of the 1301 participants are shown in Table 1. The average age was 38.86 ± 9.00 years, and there was no statistically significant difference in age between males and females (*P* = 0.074). The overall percentage of MS in the subjects was 17.4% (30.7% in males and 7.0% in females, *P* < 0.001). CAVI value and the other variables were significantly higher in males than in females, with the exception of HDL-C and the percentage of low HDL-C.

**Table 1:** Baseline characteristics of subjects.

Variables	All (N = 1301)	Male (n = 573)	Female (n = 728)	*P* value
Age (years)	38.86 ± 9.00	39.36 ± 8.98	38.46 ± 9.01	0.074
Current smoker, n (%) *	272 (20.9)	263 (45.9)	9 (1.2)	< 0.001
Current drinker, n (%) *	503 (38.7)	374 (65.3)	129 (17.7)	< 0.001
High-stress life, n (%) *	407 (31.3)	201 (35.1)	206 (28.3)	0.009
BMI (kg/m^2^) *	23.86 ± 3.78	25.47 ± 3.48	22.59 ± 3.52	< 0.001
SBP (mmHg) *	120.27 ± 15.77	128.17 ± 14.84	114.05 ± 13.55	< 0.001
DBP (mmHg) *	75.59 ± 10.97	80.77 ± 10.27	71.51 ± 9.71	< 0.001
FPG (mmol/L) *	5.12 ± 1.01	5.33 ± 1.19	4.96 ± 0.81	< 0.001
WC (cm) *	80.28 ± 10.89	87.91 ± 9.37	74.27 ± 7.80	< 0.001
TG (mmol/L) *	1.24 (0.86, 1.99)	1.74 (1.19, 2.55)	0.98 (0.75, 1.44)	< 0.001
HDL-C (mmol/L) *	1.51 ± 0.35	1.34 ± 0.29	1.64 ± 0.33	< 0.001
Non-HDL (mmol/L) *	3.37 ± 0.94	3.70 ± 0.93	3.11 ± 0.86	< 0.001
AIP *	- 0.07 (-0.29, 0.18)	0.14 (-0.08, 0.33)	- 0.22 (-0.37, -0.02)	< 0.001
AC *	2.39 ± 0.94	2.89 ± 0.94	1.99 ± 0.72	< 0.001
Coronary heart disease, n (%)	33 (2.5)	18 (3.1)	15 (2.1)	0.218
Stroke, n (%)*	5 (0.4)	5 (0.9)	0	0.012
High BP, n (%) *	367 (28.2)	260 (45.4)	107 (14.7)	< 0.001
High WC, n (%) *	525 (40.4)	351 (61.3)	174 (23.9)	< 0.001
High TG, n (%) *	429 (33.0)	295 (51.5)	134 (18.4)	< 0.001
Low HDL-C, n (%) *	155 (11.9)	50 (8.7)	105 (14.4)	0.002
High FPG, n (%) *	161 (12.4)	112 (19.5)	49 (6.7)	< 0.001
MS, n (%) *	227 (17.4)	176 (30.7)	51 (7.0)	< 0.001
CAVI *	7.46 ± 0.83	7.54 ± 0.88	7.40 ± 0.78	0.003

*P* values were calculated by Student’s t-test or *χ*^*2*^-test.

Values are shown as the mean ± SD or median (interquartile range) for continuous data and number and proportions for categorical data.

Abbreviations: BMI, body mass index; SBP, systolic blood pressure; DBP, diastolic blood pressure; WC, waist circumference; TG, triglyceride; HDL-C, high-density lipoprotein cholesterol; AIP, atherogenic index of plasma; AC, atherogenic coefficient; FPG, fasting plasma glucose; BP, blood pressure; MS, metabolic syndrome; CAVI, cardio-ankle vascular index.

* *P* value < 0.05 between males and females.

Table 2 shows the differences in MS and non-MS subjects stratified by gender. CAVI showed a statistically significant difference in female subjects with and without MS, but not in males. Moreover, there were significant differences in all other variables except for the percentage of drinking and high-stress life in both genders.

**Table 2:** Characteristics of subjects stratified by sex and the presence/absence of metabolic syndrome.

Variables	Male (n = 573)		Female (n = 728)	
	Non-MS (n = 397)	MS (n = 176)	Non-MS (n = 677)	MS (n = 51)
Age (years) *^Δ^	38.68 ± 8.87	40.90 ± 9.05	37.92 ± 8.96	45.63 ± 6.25
Current smoker, n (%) *^Δ^	170 (42.8)	93 (52.8)	6 (0.9)	3 (5.9)
Current drinker, n (%)	253 (63.7)	121 (68.8)	123 (18.2)	6 (11.8)
High-stress life, n (%)	142 (35.8)	59 (33.5)	191 (28.2)	15 (29.4)
BMI (kg/m^2^) *^Δ^	24.51 ± 3.12	27.63 ± 3.31	22.29 ± 3.35	26.48 ± 3.42
SBP (mmHg) *^Δ^	123.32 ± 13.40	139.10 ± 11.84	112.69 ± 12.12	132.20 ± 17.95
DBP (mmHg) *^Δ^	77.71 ± 9.48	87.68 ± 8.50	70.60 ± 8.94	83.57 ± 11.47
FPG (mmol/L) *^Δ^	5.08 ± 0.81	5.90 ± 1.63	4.89 ± 0.50	5.97 ± 2.25
WC (cm) *^Δ^	84.98 ± 8.49	94.51 ± 7.79	73.53 ± 7.26	84.06 ± 8.18
TG (mmol/L) *^Δ^	1.40 (1.05, 1.99)	2.59 (2.04, 3.67)	0.95 (0.73, 1.34)	2.20 (1.77, 2.87)
HDL-C (mmol/L) *^Δ^	1.40 ± 0.28	1.21 ± 0.25	1.67 ± 0.32	1.23 ± 0.23
Non-HDL-C (mmol/L) *^Δ^	3.53 ± 0.91	4.10 ± 0.87	3.07 ± 0.85	3.71 ± 0.79
AIP *^Δ^	0.01 (-0.16, 0.19)	0.33 (0.20, 0.53)	- 0.23 (-0.38, -0.06)	0.27 (0.12, 0.43)
AC *^Δ^	2.62 ± 0.83	3.51 ± 0.88	1.91 ± 0.65	3.09 ± 0.77
Coronary heart disease, n (%) ^Δ^	15 (3.8)	3 (1.7)	11 (1.6)	4 (7.8)
Stroke, n (%)	3 (0.8)	2 (1.1)	0	0
High BP, n (%) *^Δ^	108 (27.2)	152 (86.4)	74 (10.9)	33 (64.7)
High WC, n (%) *^Δ^	182 (45.8)	169 (96.0)	129 (19.1)	45 (88.2)
High TG, n (%^)^ *^Δ^	132 (33.2)	163 (92.6)	92 (13.6)	42 (82.4)
Low HDL-C, n (%) *^Δ^	11 (2.8)	39 (22.2)	65 (9.6)	40 (78.4)
High FPG, n (%) *^Δ^	28 (7.1)	84 (47.7)	29 (4.3)	20 (39.2)
CAVI ^Δ^	7.57 ± 0.80	7.48 ± 1.04	7.37 ± 0.77	7.80 ± 0.81

*P* values were calculated by Student’s t-test or χ^2^-test.

Values are shown as the mean ± SD or median (interquartile range) for continuous data and number and proportions for categorical data. Abbreviations as in Table 1.

* *P* < 0.05 in males

^Δ^ *P* < 0.05 in females.

### The effect of age on CAVI

**Fig 1:**
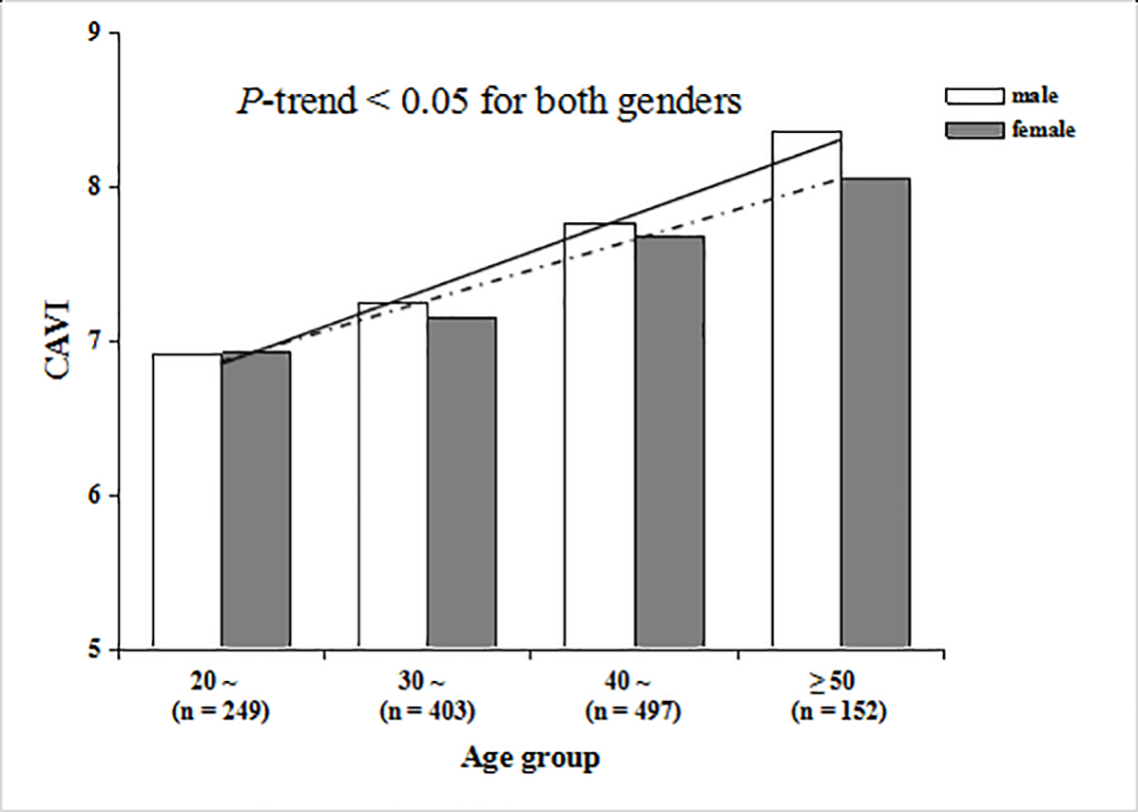
Trend test of CAVI values with age in males and females. The solid line is the trendline for males, and the dotted line is the trendline for female.

Although it is not a component of MS, age has long been considered one of the uncontrollable factors affecting arterial stiffness. Our results showed that the mean of CAVI values among different age groups was statistically significant in both sexes. The changes of CAVI with age for the two sexes are presented in Fig 1. The results showed that CAVI values increased linearly with age in both sexes (*P*-trend < 0.001 for both). Further, CAVI values in males were more influenced by age than those in females (the linear correlation coefficient was 0.502 in males and 0.477 in females).

### Relationship between the indexes of plasma atherogenicity and MS components

**Fig 2:**
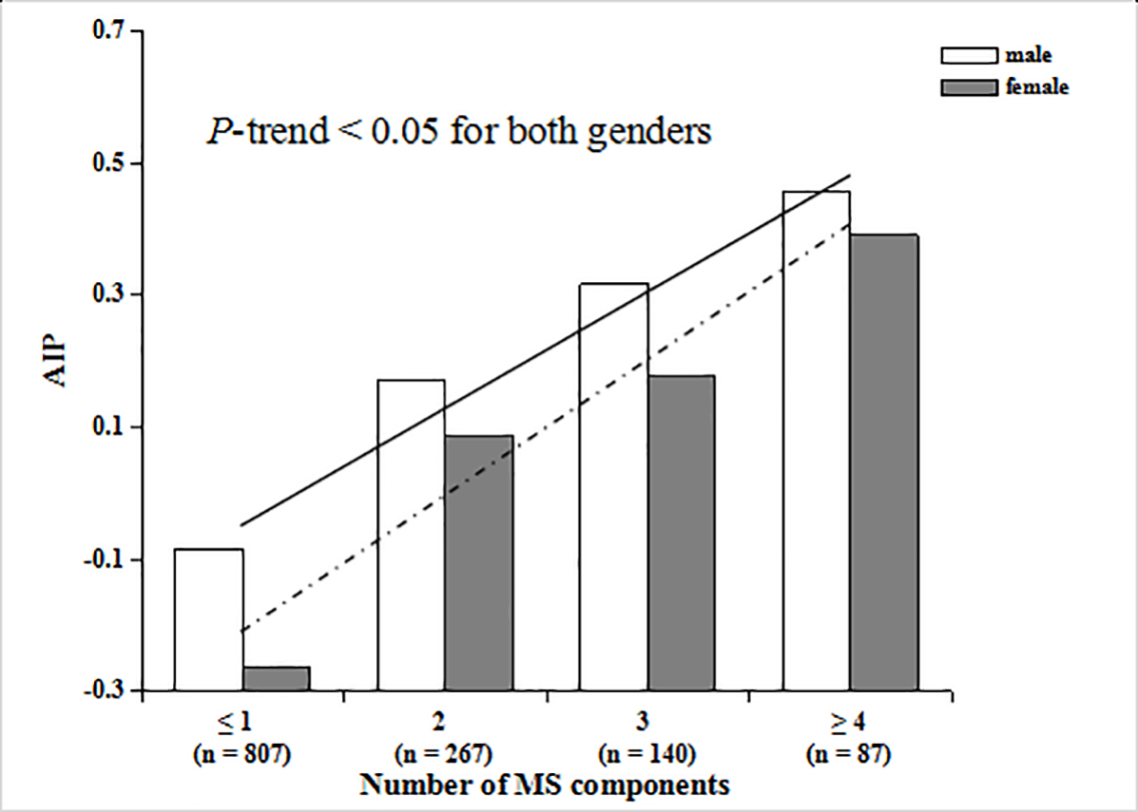
Trend test of AIP with the number of MS components in males and females. The solid line is the trendline for males, and the dotted line is the trendline for females.

**Fig 3:**
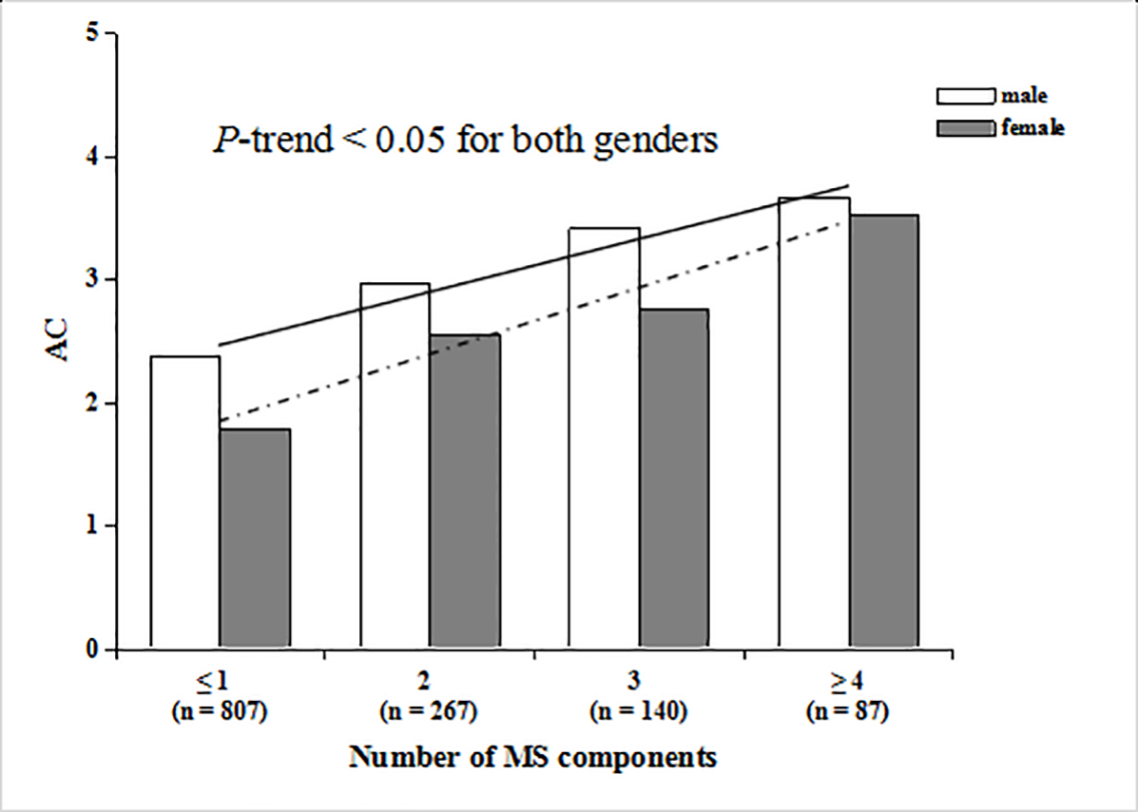
Trend test of AC with the number of MS components in males and females. The solid line is the trendline for males, and the dotted line is the trendline for females.

Figs 2 and 3 show the associations of the number of MS components with AIP and AC, respectively. We found that AIP and AC were significantly and positively correlated with the number of MS components in both genders, and the correlations were linear (*P*-trend < 0.05 for both).

### Relationship between MS components and CAVI

**Fig 4:**
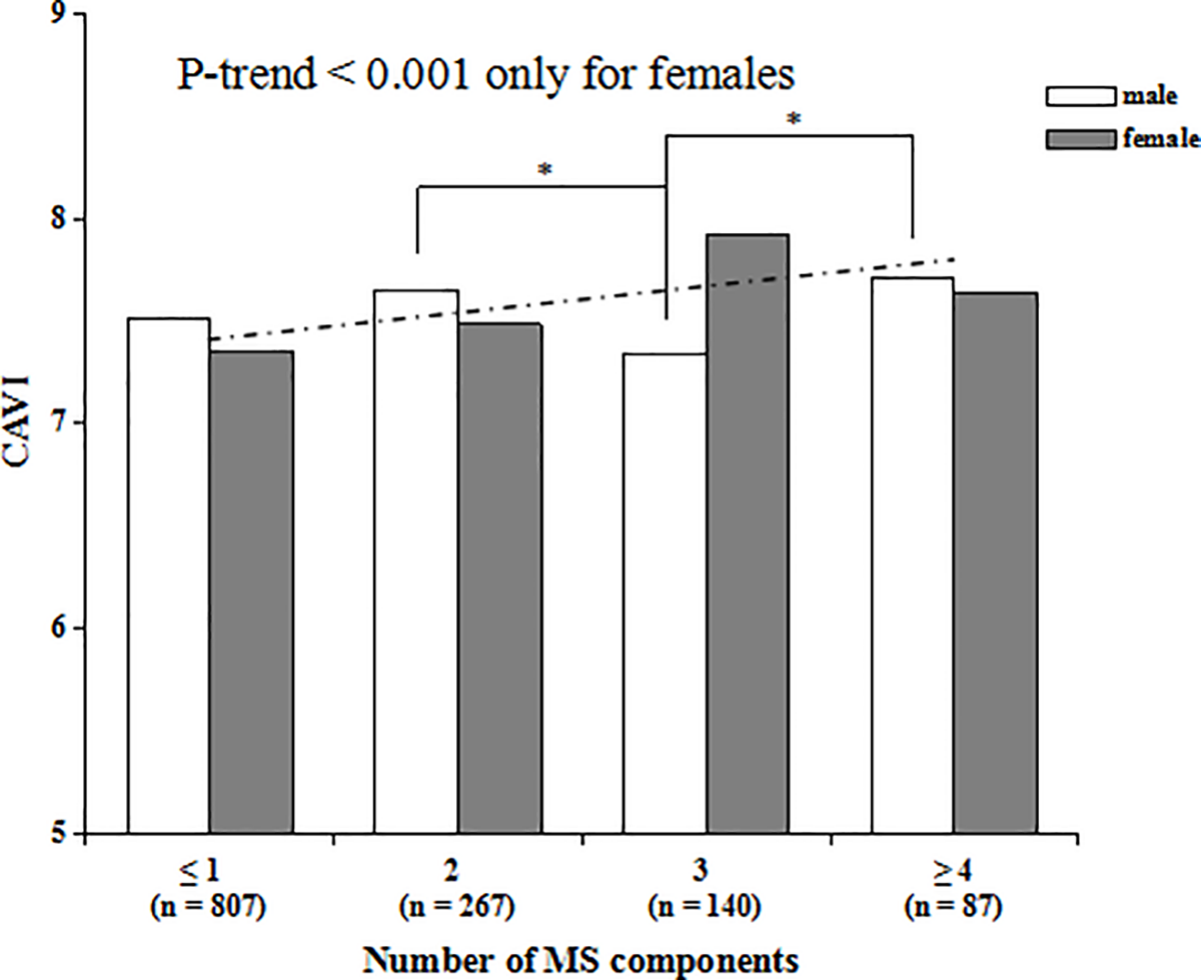
Trend test of CAVI values with the number of MS components in males and females. The solid lines show the differences between the groups in males, and the dotted line is the trendline for females. * *P* < 0.05 in males.

Fig 4 shows the correlation of the number of MS components with CAVI values. The results show that CAVI values increased linearly with the number of MS components added in females (*P*-trend < 0.001), while this trend did not exist in males (*P*-trend = 0.427).

Pearson’s correlation analysis showed that BMI and all MS components were associated with CAVI, except for WC and HDL-C. Among males, BMI, DBP, FPG, and WC were associated with CAVI, while SBP, DBP, and FPG were associated with CAVI in females (Table 3). In the multivariate analysis, BMI was negatively correlated with CAVI in the overall population and in both genders. FPG and HDL-C were associated with CAVI in the overall population and HDL-C was associated with CAVI in males. However, SBP, DBP, TG and WC were not correlated with CAVI, which may be adjusted by other variates such as age, smoking and drinking status, high-stress life and BMI in multivariate linear analysis (Table 4).

**Table 3:** Pearson’s correlation coefficients between MS components and CAVI.

Variables	All (N = 1301)		Male (n = 573)		Female (n = 728)	
	r	P value	r	P value	r	P value
BMI	- 0.060	0.031*	- 0.211	< 0.001**	0.004	0.904
SBP	0.083	0.003**	- 0.020	0.638	0.122	0.001**
DBP	0.138	< 0.001**	0.083	0.046*	0.145	< 0.001**
FPG	0.161	< 0.001**	0.170	< 0.001**	0.124	0.001**
WC	0.003	0.912	- 0.124	0.003**	0.006	0.872
TG	0.070	0.012*	0.031	0.457	0.066	0.074
HDL-C	- 0.025	0.365	0.033	0.427	- 0.006	0.863
Number of MS components	0.092	0.001**	0.014	0.743	0.138	< 0.001**

Abbreviations as in Table 1.

* *P <*0.05

** *P <*0.01.

**Table 4:** Multiple linear regression models evaluating the associations of MS components and CAVI.

Variables	All (N = 1301)		Male (n = 573)		Female (n = 728)	
	β	P value	β	P value	β	P value
BMI	- 0.057	< 0.001**	- 0.061	< 0.001**	- 0.043	< 0.001**
SBP	- 0.001	0.612	0.001	0.736	- 0.005	0.188
DBP	0.003	0.441	- 0.001	0.928	0.003	0.479
FPG	0.044	0.036*	0.054	0.059	0.024	0.464
WC	0.000	0.900	- 0.001	0.809	- 0.007	0.145
TG	- 0.018	0.423	- 0.005	0.849	- 0.064	0.083
HDL-C	- 0.171	0.012*	- 0.277	0.031*	- 0.018	0.823
Number of MS components	0.014	0.677	- 0.028	0.569	0.134	0.012*

Multiple linear regression models were used to analyze the association of MS components and CAVI values, both globally and stratified by gender. Age, smoking and drinking status, high-stress life, BMI, non-HDL, coronary heart disease, and stroke were adjusted in the regression models. Abbreviations as in Table 1.

* *P* <0.05

** *P* <0.01.

## Discussion

Old age, obesity, hypertension, dyslipidemia and dysglycemia are risk factors of coronary artery disease [5]. In addition to age, the combination of these factors constitutes metabolic syndrome. Consequently, MS may increase the risk of coronary artery disease. Therefore, the early detection and management of metabolic syndrome and its components is of great importance in preventing atherosclerosis and cardiovascular events caused by atherosclerosis [25].

CAVI, the measurement of which is noninvasive, quick and convenient and has the advantage of not being affected by blood pressure, is a reliable predictor of arterial stiffness and early arteriosclerosis. Therefore, we evaluated the association of MS components with CAVI as the main marker of arterial stiffness in this study.

Age has long been thought to be a significant risk factor for arterial stiffness, directly influencing the values of CAVI [21, 26]. In this study, we found that CAVI increases linearly with age in both genders. Furthermore, CAVI values of males were higher than those of females in almost all age groups, and the increase of CAVI with age was more significant in males.

In this cross-sectional study, the prevalence of MS and all its components, except for low HDL-C, were significantly higher in males than in females, and the values of BP, WC, FPG and TG were also higher in males than in females, which are findings that were in accord with the population-based study from Chinese Center For Disease Control and Prevention (CDC) of the prevalence of metabolic syndrome in Chinese adults [27]. In the Chinese population, BP, WC, FPG and TG are usually higher in males than in females. In our study, the risk factors (including smoking, drinking and high-stress life) were more common in males than in females (Table 1), which may be mainly responsible for this phenomenon.

Consistent with the results of previous studies [28, 29], the CAVI values of males were significantly higher than that of females. First, the primary reason for this may be the influence of estrogen, which has a protective effect on blood vascular [30, 31]. Unfortunately, we failed to obtain relevant data in this study. We will further investigate the role of estrogen in the association of MS with CAVI in our further studies. Second, social and environmental factors are another important cause for this difference. In China, smoking, drinking and a high-stress life are more common in males than in females, and these factors have been proven to accelerate the progression of vascular sclerosis [32]. After grouping by the presence of MS, CAVI values in women with MS were significantly higher than those in non-MS subjects, while this difference did not exist in men. As a significant factor of CAVI, age may explain this phenomenon. In the total number of subjects, there was no difference in age between males and females (39.36 ± 8.98 vs. 38.46 ± 9.01; *P* = 0.074). However, the average age of male subjects with MS was younger than that of females with MS (40.90 ± 9.05 vs. 45.63 ± 6.25; *P* < 0.001). This finding suggests that arterial stiffness develops gradually with age, and perhaps having MS at a relatively young age does not significantly influence the progression of atherosclerosis. Therefore, it is necessary to focus attention on early detection of and intervention for MS to reduce the impairment it causes to artery walls.

In this study, we have investigated the association of MS and its components with CAVI. The results showed that FPG and HDL-C were related to CAVI overall. HDL-C was associated with CAVI in men, while the number of MS components was associated with CAVI in women. It has been confirmed that each MS component has an obvious sex-dependent effect on PWV [33], while the gender-specific differences in the correlation of MS components with CAVI are not clear. The differences in biology and physiology between men and women may explain these gender differences. Further studies are needed to explore the specific mechanisms of these differences.

Nam et al. reported that CAVI increases with increasing FPG [34]. Other studies have reached a similar conclusion that FPG is positively correlated with CAVI [21]. Researchers have found that hyperglycemia leads to many alterations in vascular tissue cells, which may accelerate the progress of atherosclerosis [29, 35]. Elevated FPG accelerating inflammation through the promotion of cytokine secretion by monocytes and adipocytes is another mechanism for inflammation reaction that plays an important role in the development of atherosclerosis [36, 37].

HDL-C has traditionally been considered as a protective factor for arteriosclerosis and cardiovascular diseases. Conclusions of previous studies that CAVI significantly increased in low HDL-C groups support this view [21, 29, 38]. The results of this study showed that HDL-C is negatively associated with CAVI in men. However, Sorrentino et al. have found that the endothelial-protective function of HDL-C in diabetics and MS patients was greatly impaired compared with healthy people [39]. This finding shows that the protective effects on endothelial function of HDL-C might not exist with the presence of diabetes or MS. An alternative view is that the correlation between HDL-C and cardiovascular diseases may be determined by size, suggested by discoveries that small-sized HDL-C is significantly associated with the risk of cardiovascular events, while the medium-sized and large-sized HDL-C are not [40, 41]. Owing to the limited data, we could not investigate the mechanisms of HDL-C in the progression of arterial stiffness at the molecular level, which provides a new direction for future research.

In our study, SBP and DBP were associated with CAVI in univariate analysis, but not in multivariate analysis, which may be affected by the adjusting variables. Raised BP directly affects the arterial walls, resulting in the increase of vascular tension. Continuous elevation of BP will lead to structural and functional changes in the arterial walls, resulting in further decrease of large arterial elasticity, which reflects arteriosclerosis.

WC reflects the amount of intra-abdominal adipose tissue, and it has been demonstrated to predict the risk of cardiovascular events and has been applied to clinical examinations [42]. As the marker of central obesity, WC has been proven to be a risk factor for arterial stiffness [16]. However, we found that WC was negatively correlated with CAVI in men in this study, which was inconsistent with the results of previous studies [16, 21, 43]. Different age composition and adjustment variables used in various research studies may result in this difference. The different association of WC with CAVI in men and women may be due to the physiological differences in body fat, which is an important factor influencing the effects of WC on arterial stiffness [16]. Further studies are needed to confirm this view.

In addition, we have found a negative correlation between BMI and CAVI in the overall population and in both genders. Nagayama et al. found that an inverse correlation between BMI and CAVI in healthy middle-aged subjects, and they hypothesized that systemic accumulation of adipose tissue, per se, leads to reduced arterial stiffness [44]. In another study of middle-aged and elderly Chinese subjects, an inverse correlation between BMI and CAVI was also observed [21]. It seems that high BMI values do not always mean high arterial stiffness, which is probably related to the distribution of body fat, as the absolute amount of adipose tissue does not increase the risk of cardiovascular disease in many overweight people [44, 45].

MS components often exist simultaneously, interacting with each other, and they may increase the risk of arterial stiffness [9]. Research has shown that CAVI values increase with each additional component of MS, even when the total number of MS components were not sufficient for the diagnosis of MS [46]. In this present study, the two indexes of plasma atherogenicity, AIP and AC, were positively correlated with the number of MS components in both males and females, which supports the view that metabolic syndrome plays an important role in the development of arterial stiffness from another aspect. Consequently, the early management of MS components is very important and could effectively decrease the risk of atherosclerosis and cardiovascular events [25].

In this study, CAVI increased linearly as the number of MS components increased in females, but not in males. This result was not consistent with that reported by Scuteri et al. [47], who found the effect of MS on arterial stiffness to be similar in both genders. Furthermore, one study on Caucasian subjects showed that the association between the number of MS components and arterial stiffness is greater in males [29]. However, several studies on Asian populations have reported that CAVI increased proportionally with the number of MS components only in females [28, 30, 43]. The primary cause for these discrepancies may be the ethnic differences and the different indexes of arterial stiffness used by these studies. Another important reason is the confounding effect of age, which could not be eliminated in this study because of the strong interaction between the number of MS components with age.

In addition, among the 728 female subjects in this study, only 51 subjects (7.0%) had MS, which may have led to a false negative association because of sample size limitations. This incidence is lower than that of the general population in China [48]. The difference of age distribution may be the main reason for this phenomenon. Our study involved 728 female subjects aged 20 to 60, their average age was 45.63 ± 6.25, and there were only 76 subjects over 50 (S1 Table). The average age and the ratio of subjects over 50 were lower than those in other researches [28, 30]. According to a meta-analysis of the prevalence of metabolic syndrome in mainland China [48], the prevalence of MS increases with age in females, peaking in the ≥ 60 years group. Further study with larger sample sizes and a broader age range is needed to clarify the positive association in females in our study.

Strengths of this study include its gender-specific exploration of the relationship between MS and CAVI, as well as its application of two lipid ratios indicating arterial stiffness to some extent. However, there remain limitations in this study as well. First, our results do not establish causal relationships between MS and CAVI because of the cross-sectional study design. Second, since all subjects were recruited from Jilin Province in China, caution should be used when extrapolating these conclusions to other regions.

## Conclusions

In conclusion, our findings suggest that MS is significantly associated with CAVI, and there are gender-specific differences in this association. The effects of the number of MS components on CAVI are stronger in females than in males, and the association of each MS component with CAVI may differ in relation to gender. Furthermore, aging affects arterial stiffness more severely in males, and the increase in arterial stiffness tends to occur at a younger age in males. We should account for gender differences into consideration when interpreting study results. Larger samples and longitudinal studies are needed to further confirm our results in the future.

## Supporting information

S1 Database The database for this study. (SAV)

S1 Table Prevalence of metabolic syndrome in different age groups stratified by gender. (DOCX)
