# Supplementary material for: Gender-specific association of metabolic syndrome and its components with arterial stiffness in the general Chinese population
Source: PLoS One. 2017 Oct 26;12(10):e0186863. doi: 10.1371/journal.pone.0186863 (PMC5658088; doi:10.1371/journal.pone.0186863)
Supplement: S1 Table — (DOCX) [file pone.0186863.s002.docx]

**S1 Table. Prevalence of metabolic syndrome in different age groups stratified by gender**

| Variables | All | Prevalence  (%) | Male | Prevalence  (%) | Female | Prevalence  (%) |
| --- | --- | --- | --- | --- | --- | --- |
| Age group |  |  |  |  |  |  |
| 20~ | 20/249 | 8.0 | 18/94 | 19.1 | 2/155 | 1.3 |
| 30~ | 64/403 | 15.9 | 61/186 | 32.8 | 3/217 | 1.4 |
| 40~ | 97/497 | 19.5 | 63/217 | 29.0 | 34/280 | 12.1 |
| ≥ 50 | 46/152 | 30.3 | 34/76 | 44.7 | 12/76 | 15.8 |
| Total | 227/1301 | 17.4 | 176/573 | 30.7 | 51/728 | 7.0 |
